# Integrase Defective Lentiviral Vector as a Vaccine Platform for Delivering Influenza Antigens

**DOI:** 10.3389/fimmu.2018.00171

**Published:** 2018-02-05

**Authors:** Alessandra Gallinaro, Martina Borghi, Roberta Bona, Felicia Grasso, Laura Calzoletti, Laura Palladino, Serena Cecchetti, Maria Fenicia Vescio, Daniele Macchia, Valeria Morante, Andrea Canitano, Nigel Temperton, Maria Rita Castrucci, Mirella Salvatore, Zuleika Michelini, Andrea Cara, Donatella Negri

**Affiliations:** ^1^National Center for Global Health, Istituto Superiore di Sanità, Rome, Italy; ^2^Department of Infectious Diseases, Istituto Superiore di Sanità, Rome, Italy; ^3^VisMederi S.r.l., Siena, Italy; ^4^Confocal Microscopy Unit NMR, Confocal Microscopy Area Core Facilities, Istituto Superiore di Sanità, Rome, Italy; ^5^Center for Animal Research and Welfare, Istituto Superiore di Sanità, Rome, Italy; ^6^Viral Pseudotype Unit, Medway School of Pharmacy, University of Kent, Kent, United Kingdom; ^7^Department of Medicine, Weill Cornell Medical College, New York, United States

**Keywords:** lentiviral vector, influenza, vaccine, antibody, T cell response

## Abstract

Viral vectors represent an attractive technology for vaccine delivery. We exploited the integrase defective lentiviral vector (IDLV) as a platform for delivering relevant antigens within the context of the ADITEC collaborative research program. In particular, Influenza virus hemagglutinin (HA) and nucleoprotein (NP) were delivered by IDLVs while H1N1 A/California/7/2009 subunit vaccine (HAp) with or without adjuvant was used to compare the immune response in a murine model of immunization. In order to maximize the antibody response against HA, both IDLVs were also pseudotyped with HA (IDLV-HA/HA and IDLV-NP/HA, respectively). Groups of CB6F1 mice were immunized intramuscularly with a single dose of IDLV-NP/HA, IDLV-HA/HA, HAp alone, or with HAp together with the systemic adjuvant MF59. Six months after the vaccine prime all groups were boosted with HAp alone. Cellular and antibody responses to influenza antigens were measured at different time points after the immunizations. Mice immunized with HA-pseudotyped IDLVs showed similar levels of anti-H1N1 IgG over time, evaluated by ELISA, which were comparable to those induced by HAp + MF59 vaccination, but significantly higher than those induced by HAp alone. The boost with HAp alone induced an increase of antibodies in all groups, and the responses were maintained at higher levels up to 18 weeks post-boost. The antibody response was functional and persistent overtime, capable of neutralizing virus infectivity, as evaluated by hemagglutination inhibition and microneutralization assays. Moreover, since neuraminidase (NA)-expressing plasmid was included during IDLV preparation, immunization with IDLV-NP/HA and IDLV-HA/HA also induced functional anti-NA antibodies, evaluated by enzyme-linked lectin assay. IFNγ-ELISPOT showed evidence of HA-specific response in IDLV-HA/HA immunized animals and persistent NP-specific CD8+ T cell response in IDLV-NP/HA immunized mice. Taken together our results indicate that IDLV can be harnessed for producing a vaccine able to induce a comprehensive immune response, including functional antibodies directed toward HA and NA proteins present on the vector particles in addition to a functional T cell response directed to the protein transcribed from the vector.

## Introduction

Improvements in existing delivery systems are a significant aspect to be considered in order to obtain more effective vaccines. Viral vectors represent an attractive platform for vaccine development due to their ability to effectively deliver antigens of interest into cells and to generate humoral and cellular mediated immune responses against the encoded transgenes. Integrase defective lentiviral vectors (IDLVs) represent a promising platform for immunogen delivery for vaccination purposes (1, 2). IDLVs are self-inactivating (SIN), non-integrating, and non-replicating vectors with high transduction efficiency both *in vitro* and *in vivo*. In contrast to parental lentiviral vector (LV), IDLVs are produced by incorporating a mutated form of the integrase (IN) protein in the recombinant LV, preventing integration and overcoming the risk of insertional mutagenesis. The loss of integration has been demonstrated in several murine models *in vivo* and *in vitro* (3). In the absence of integration, transgene expression is due to the unintegrated circular forms of the vector, which are maintained episomally in the target cells in the absence of cell division (4, 5). Only the transgene of interest is expressed from episomal IDLV in the absence of any other parental viral product. Dendritic cells and macrophages, the main cell types mediating the immune response, are non-dividing cells that are readily transduced by IDLV, eliciting the expansion of antigen-specific T cells (6, 7).

Over the course of the past decade, several reports have shown that a single immunization with IDLV-vectored antigens induces a persistent immune response both in murine and in simian models of immunization (1, 2, 8). Antigen presentation persisted for at least 30 days from immunization (9), suggesting that prolonged expression might be a unique characteristic of IDLV *in vivo*. In preclinical challenge experiments, a single immunization with IDLV expressing human papillomavirus-E7 tumor-specific antigen resulted in eradication of established tumors in mice, validating the ability to induce an effective T cell response (10). Administration of IDLV encoding antigens in murine models of West Nile Virus and malaria induced protective antibodies when challenged with the respective pathogens (11, 12). IDLV expressing the influenza virus nucleoprotein (NP) was protective against homologous and heterosubtypic influenza virus challenge (13). More recently we showed that immunization of *rhesus macaques* with IDLV expressing HIV-Env 1086.C gp140 induced broad and sustained immune responses up to 1 year from the immunization (8). Importantly, IDLV is under evaluation in clinical trials for cancer immunotherapy (ClinicalTrials.gov Identifier numbers: NCT02609984, NCT02122861, NCT02387125).

In addition to the potential for inducing a prolonged immune response due to expression of the vectored transgene from episomal DNA circles, IDLV can be harnessed as a cargo for delivering immunogens after incorporation into the vector’s particles. This can be accomplished by fusion of foreign antigens with proteins incorporated into the lentiviral particles during particle assembly (14, 15) or *via* pseudotyping. Pseudotyping with heterologous viral glycoprotein envelopes is always used during LV production for allowing transduction of target cells or tissues (16). LV particles can be pseudotyped with a wide range of heterologous viral envelope proteins, including Influenza virus hemagglutinin (HA) (17–19). Recovered particles gain the tropism of the virus from which the envelope glycoprotein was derived. The most widely used envelope glycoprotein for pseudotyping LV is the vesicular stomatitis G glycoprotein (VSV.G), which allow broad and efficient transduction of target cells *in vitro* and *in vivo* (20). Importantly, the envelope glycoprotein displayed on the surface of the particles can elicit humoral immune responses that can be protective in animal models of immunizations (21–23).

Seasonal influenza A virus (IAV) infections cause significant morbidity and mortality worldwide and remain a major public health concern (24, 25). Currently licensed influenza vaccines elicit neutralizing antibodies (Abs) targeting HA, preventing influenza virus entry into cells (26). In particular, HAs from influenza A (H1N1) pdm09 virus circulating in humans are a major antigenic component contained in the annual vaccine formulations (27). However, seasonal vaccines do not protect against new mismatched strains and require frequent reformulation based on the prediction of strains that may circulate (27). Conversely, cell-mediated immunity targeting conserved antigens, such as influenza NP, is cross reactive and, although T cell immunity is unable to prevent disease, may contribute to improve clearance and decreased symptoms (28–30). NP is 90% conserved among influenza virus strains (31), and it is the major target of the cross-protective T cell response against influenza virus in the mouse model (32–35). However, while protection from influenza challenge in mice can be achieved in presence of NP-specific T cell responses (36), an efficient influenza vaccine for humans should be able to generate a more comprehensive and durable immune response in terms of both protective antibodies and effective T cells.

To this aim, in the present study, we evaluated the immune response induced by a multi-antigen IDLV-based influenza vaccine pseudotyped with HA protein from A/California/7/09 (H1N1-pdm09) virus and expressing either influenza HA or NP proteins as transgenes. Results indicated that a single immunization with multivalent IDLVs induced persistent and effective antiviral Abs and T cell responses directed toward HA or NP transgenes.

## Materials and Methods

### Vector Construction

The SIN lentiviral transfer vector plasmid pTY2-NP expressing the nucleprotein (NP, GenBank: AAM75159.1) from Influenza virus A/PR/8/1934 (H1N1) has been already described (13). For construction of SIN lentiviral transfer vector plasmid pGAE-HA expressing the hemagglutinin (HA, GenBank: ACP44189.1) from influenza virus A/California/7/2009 (H1N1), the codon optimized HA open reading frame (ORF) was chemically synthesized, inserted into pUC57 plasmid (TwinHelix, Milan, Italy), excised with AgeI/SalI restriction enzymes and cloned into pGAE-green fluorescent protein (GFP) lentiviral transfer vector (37) by replacing the GFP coding sequence. The IN defective packaging plasmid, producing the viral proteins necessary for vector particle production, and the phCMV-VSV.G plasmid, encoding the pseudotyping vesicular stomatitis virus envelope glycoprotein G (VSV.G) from Indiana serotype, necessary for IDLV entry into target cells, have been previously described (38, 39). Plasmids pCAGGS-TMPRSS2 and pCAGGS-HAT, expressing the transmembrane protease serine 2 (TMPRSS2, GenBank: U75329) and the human airway trypsin (HAT, GenBank: AB002134) proteins, respectively, were described previously (40). Plasmids pCMV3-H1N1-C-NA (Sino Biological Inc., North Wales, PA, USA), expressing the neuraminidase (NA, GenBank: ACP41107.1) derived from influenza virus A/California/4/2009 (H1N1), and pCMV-HACal09, expressing the codon optimized HA protein from the CMV promoter, were used during production of HA-pseudotyped IDLV-NP/HA.

### Production of Lentiviral Vectors

Lenti-X human embryonic kidney 293T cell line was obtained from Clontech (Mountain View, CA, USA) and was used for IDLV production following established protocols (41). Cells were maintained in Dulbecco’s modified eagles medium (Gibco, Life Technologies Italia, Monza, Italy) supplemented with 10% fetal calf serum (Corning, Mediatech Inc., Manassas, VA, USA) and 100 units/ml penicillin/streptomycin/glutamine (PSG) (Gibco, Life Technologies Italia, Monza, Italy). For production of IDLVs, pseudotyped with HA and expressing either HA (IDLV-HA/HA) or NP (IDLV-NP/HA), 3.5 × 10^6^ 293 T Lenti-X cells were seeded on 10-cm Petri dishes (Corning Incorporated—Life Sciences, Oneonta, NY, USA) and incubated overnight. Cells were transiently transfected with lentiviral transfer vector expressing influenza HA or NP, along with IN defective packaging and VSV.G-envelope plasmids by Calcium Phosphate using the Profection Mammalian Transfection System (Promega Corporation, Madison, WI, USA) as previously described (13, 39, 41). Plasmids pCAGGS-TMPRSS2 or pCAGGS-HAT and plasmid pCMV3-H1N1-C-NA were included to express protease and NA during IDLV production. To pseudotype IDLV-NP/HA with HA protein, pCMV-HACal09 plasmid was added during IDLV-NP/HA production. After 48 and 72 h post-transfection, cell culture supernatants were collected, cleared from cellular debris by low-speed centrifugation and passed through a 0.45-µm pore size filter (Millipore Corporation, Billerica, MA, USA). To produce IDLV stocks for mouse immunization, vector containing supernatants were concentrated by ultracentrifugation (Beckman Coulter, Fullerton, CA, USA) on a 20% sucrose gradient (Sigma Chemical Co., St. Louis, MO, USA) at 23.000 rpm for 2.5 h at 4°C using a SW28 swinging bucket rotor (Beckman). Pelleted vector particles were resuspended in 1× phosphate-buffered saline (PBS, Gibco, Life Technologies Italia, Monza, Italy) and stored at −80°C until use. Each IDLV-HA/HA or IDLV-NP/HA stock was titred by the reverse transcriptase (RT) activity assay and the corresponding transducing units (TU) were calculated by comparing the RT activity to the one of IDLV-GFP virions with known infectious titers, thus allowing for the determination of their infectious titer units (42).

### Flow Cytometry and Confocal Laser Scanning Microscopy (CLSM)

293 T Lenti-X cells were plated in six-well microplates for flow cytometry analysis or seeded in 24-well cluster plates onto 12-mm cover glasses previously treated with l-polylysine (SIGMA) for CLSM. Cells were then transfected by calcium phosphate with pCMVHACal09 using the Profection Mammalian Transfection System (Promega). Twenty-four hours following transfection, non-permeabilized cells were stained with antiserum from CB6F1 mice immunized with purified H1N1 subunit vaccine (0.1 µg of HA per dose) from influenza strain H1N1 A/California/7/2009 (provided by Novartis Vaccine & Diagnostics Srl, Siena, Italy) in the presence of MF59 adjuvant (provided by Novartis). After 45 min, cells were washed in PBS 1× and then incubated for 30 min with secondary antibody PE Goat antimouse IgG (Biolegend, San Diego, CA, USA), for flow cytometry analysis, or Alexa Fluor-488-F(ab′)2 fragments of goat antimouse (Molecular Probes, Life Technologies, Carlsbad, CA, USA), for CSLM analysis. For flow cytometry, cells were fixed with 1% paraformaldehyde and fluorescence was measured using the FACSCalibur (BD Biosciences, Milan, Italy), and data were analyzed using CellQuest (BD Biosciences). For CLSM, cells were fixed with methanol and the coverslips were mounted with Vectashield^®^ antifade mounting medium containing DAPI (Vector Labs, Burlingame, CA, USA) on the microscope slides. CLSM observations were performed on a Leica TCS SP2 AOBS apparatus (Leica Microsystems, Wetzlar, Germany), using excitation spectral laser lines at 405 and 488 nm, using the confocal software (Leica, Wetzlar, Germany) and Photoshop CS5 (Adobe Systems, San Jose, CA, USA). Signals from different fluorescent probes were taken in sequential scanning mode, several fields were analyzed for each labeling condition, and representative results are shown. Images represented a single central optical section taken in the center of each cell nucleus and a 3D reconstruction.

### Western Blot

To evaluate HA presence on IDLV particles, pellets of IDLV concentrated preparations were resuspended in SDS loading buffer. Lysed virions were separated on 12% SDS polyacrylamide gel under reducing conditions and transferred to a nitrocellulose membrane (Sartorius Stedim Italy). Filters were saturated for 2 h with 5% nonfat dry milk in PBST (PBS with 0.1% Tween 20) and then incubated with HA antiserum for 1 h at room temperature followed by incubation for 1 h at room temperature with an antimouse horse radish peroxidase (HRP)-conjugated IgG (Sigma Aldrich, Milan, Italy). The immunocomplexes were visualized using chemiluminescence ECL detection system (Luminata Forte Western HRP Substrate, Millipore). H1N1 subunit vaccine from A/California/7/2009 virus was used as positive control.

### Mice and Immunization Schedule

CB6F1 female mice were purchased from Harlan (Harlan Laboratory, Srl, San Pietro al Natisone, Italy). Six- to eight-week-old CB6F1 mice, four mice per group, were injected once intramuscularly in the thigh with (i) 1 × 10^7^ RT units/mouse of IDLV-HA/HA, (ii) 1 × 10^7^ RT units/mouse of IDLV-NP/HA, (iii) H1N1 A/California/7/2009 (0.1 μg/mouse of HA protein, henceforth referred to as HAp) alone, and (iv) 0.1 μg/mouse of HAp in presence of MF59 adjuvant (HAp + MF59). Naïve, non-immunized mice were kept for parallel analysis. All immunized mice were boosted with HAp alone 24 weeks after the prime. Antibodies (Abs) were measured in serum at different time points, starting from 2 weeks after the prime and up to 18 weeks after the boost. The cellular immune responses were analyzed at 4, 12, and 24 weeks after the prime in blood samples and in splenocytes at sacrifice (18 weeks after the boost).

Serum samples were obtained from blood collected from the retro-orbital plexus of mice with glass Pasteur pipettes and stored at −20°C until assayed. Heparin-treated glass Pasteur pipettes were used to collect blood in order to perform IFNγ ELISPOT assay. Leukocytes, obtained after ammonium chloride potassium (ACK) treatment of whole blood, were counted, suspended in RPMI 1640 (Gibco) containing 10% fetal bovine serum (Lonza, Treviglio, Milan, Italy), 100 units/ml of PSG (Gibco), non-essential aminoacids (Gibco), sodium pyruvate 1 mM (Gibco), HEPES buffer solution 25 mM (Gibco), and 50 mM 2-mercaptoethanol (Sigma Chemicals). Splenocytes were prepared by mechanical disruption and passage through cell strainers (BD Biosciences) and resuspended in complete RPMI medium, as previously described (39).

## IFNγ ELISPOT

The IFNγ ELISPOT assay was performed using the BD ELISPOT kit reagents and protocol (BD Biosciences). Briefly, blood or spleen derived cells were seeded at a density of 2.5 × 10^5^/well in 96 well plates and stimulated overnight either with 2 µg/ml of the H-2Kd restricted Influenza NP_147–155_ (TYQRTRALV) epitope or with 10 µg/ml of concanavalin A (Sigma Chemicals) used as a positive control. A 139 peptide array (15mers with 11 amino acid overlaps) spanning the entire HA from influenza virus A/California/7/09 protein (BEI Resources, Manassas, VA, USA; Catalog No. NR-15433) was distributed in ten pools of 14 peptides each and used to identify the reactive epitopes on splenocytes. Complete medium treated cells were used as negative controls. Spot forming cells (SFC) were counted with an ELISPOT reader (A.EL.VIS, Hannover, Germany) and results expressed as number of IFNγ secreting cells (SFC)/10^6^ cells. The samples were scored positive when a minimum of 50 spots per 10^6^ cells were present and twofold higher than unstimulated sample.

### Measurement of Binding and Functional Antibodies

Sera were tested for the presence of binding Abs by a standard ELISA. Ninety-six well plates (Greiner bio-one, Kremsmünster, Austria) were coated with H1N1 A/California/4/09 subunit vaccine (0.2 μg/well of HA) overnight at 4°C. After washing and blocking, serial dilutions of serum from individual mice were added to wells in duplicate and incubated for 2 h at room temperature. The plates were washed and biotin-conjugated goat antimouse IgG (Southern Biotech, Birmingham, AL, USA) was added to the wells for 2 h at room temperature. The plates were washed before the addition of HRP-conjugated streptavidin (AnaSpec, Fremont, CA, USA) for 30 min at room temperature, followed by the 3,3′5,5-tetramethylbenzidine substrate (SurModics BioFX, Edina, MN, USA). Endpoint titers were determined as the reciprocal of the highest dilution giving an absorbance value at least equal to twofold that of background (biological sample from naive mice). For each group of immunization, results were expressed as mean titer with confidence interval.

Hemagglutination inhibition (HAI) Abs to A/California/7/09 virus were measured, according to standard procedures (43). Briefly, all sera were treated with receptor-destroying enzyme (Sigma Aldrich) to remove non-specific inhibitors of hemagglutination. Serial twofold dilutions of treated sera were mixed with 4 hemagglutinin units of the A/California/7/09 virus and, after 1 h incubation at room temperature, with 0.5% Turkey red blood cells. The HAI titers were expressed as the reciprocal of the highest dilution of serum that inhibited virus-induced hemagglutination.

The sera were tested by MDCK cell-based microneutralization (MN) assay (44) to titrate influenza virus-specific neutralizing antibodies. Briefly, all samples were heat-treated at 56°C for 30 min. Twofold serial dilutions of samples, starting from 1:10 dilution, were performed across the 96-well plates and then the viral working solution of A/California/07/2009 (H1N1) live virus was added (200 TCID50/100 μl). After incubation of mixture sera-virus at 37°C for 1 h, a cell suspension of 2 × 10^5^ was added and the plates were incubated at 37°C for 5 days. Each plate was then checked under an optical microscope to assess the presence of local lesions and cytopathogenic effect. The neutralization titer (NT) for each serum was calculated according to the Spearman-Kärber formula.

Since NA plasmid was included during the IDLV preparation and NA protein may be present in subunit vaccine preparations, the heat-treated sera were also tested by enzyme-linked lectin assay (ELLA) (45) to measure the functional anti-NA Abs. Briefly, serial twofold dilutions of treated sera were mixed with antigen A/California/07/2009 (H1N1) treated with Triton X-100 as described elsewhere, in 96-well plates coated with fetuin. The plates were incubated at 37°C overnight (16–18 h). After washing the plates, the peanut agglutinin conjugate with peroxidase was added and the plates were incubated at room temperature for 2 h in the dark. Then the o-phenylenediamine dihydrochloride substrate was added and the plates were incubated at room temperature for 10 min in the dark. The reaction was stopped by adding 1 N H2S04. The optical density of all the test plates was read at 490 nm. The NA inhibition (NI) titer was expressed as the reciprocal of the last dilution that results in at least 50% inhibition of the maximum signal.

### Statistical Analysis

The temporal trend of antibody response (i.e., ELISA, HAI, MN, and ELLA) was analyzed using a system of piecewise linear equations in order to jointly evaluate the relationships of each outcome at each time point, allowing for correlated errors. The analysis was conducted in STATA13 (StataCorp LP, College Station, TX, USA) within a structural equation modeling frame work (46, 47). The log likelihood estimation procedure was used to fit the model to the data.

## Results

### Production of IDLV Delivering Influenza HA

**Figure 1:**
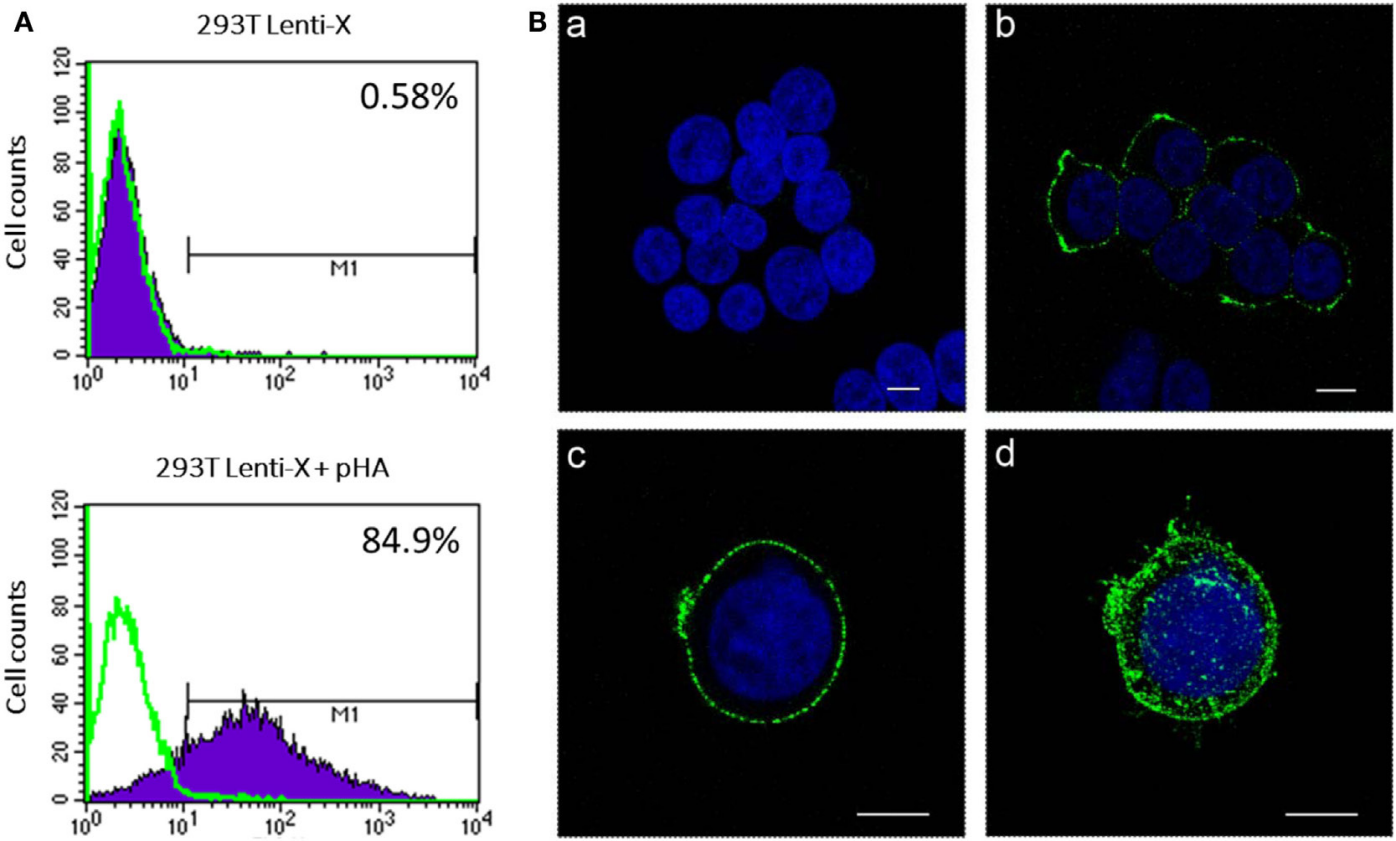
Analysis of hemagglutinin (HA) expression. 293 T Lenti-X cells were transfected with HA expressing plasmid and stained at 24 h post-transfection for detection of HA on the plasma membrane as described in Section “Materials and Methods.” Cells were fixed and expression of HA was quantitatively measured by flow cytometry **(A)** or observed by confocal laser scanning microscopy **(B)**. **(A)** The percentage of HA-expressing cells is indicated. The overlay line (green) represents the fluorescence distribution of cells stained only with the secondary antibody. **(B)** Images represent single central optical sections (a–c) and a 3D reconstruction (d). Nuclei are colored in blue by DAPI staining and green color represents membrane associate HA protein. Scale bars, 8 µm. Untransfected cells (a) were used as negative control. Results from one representative experiment are shown for each analysis.

**Figure 2:**
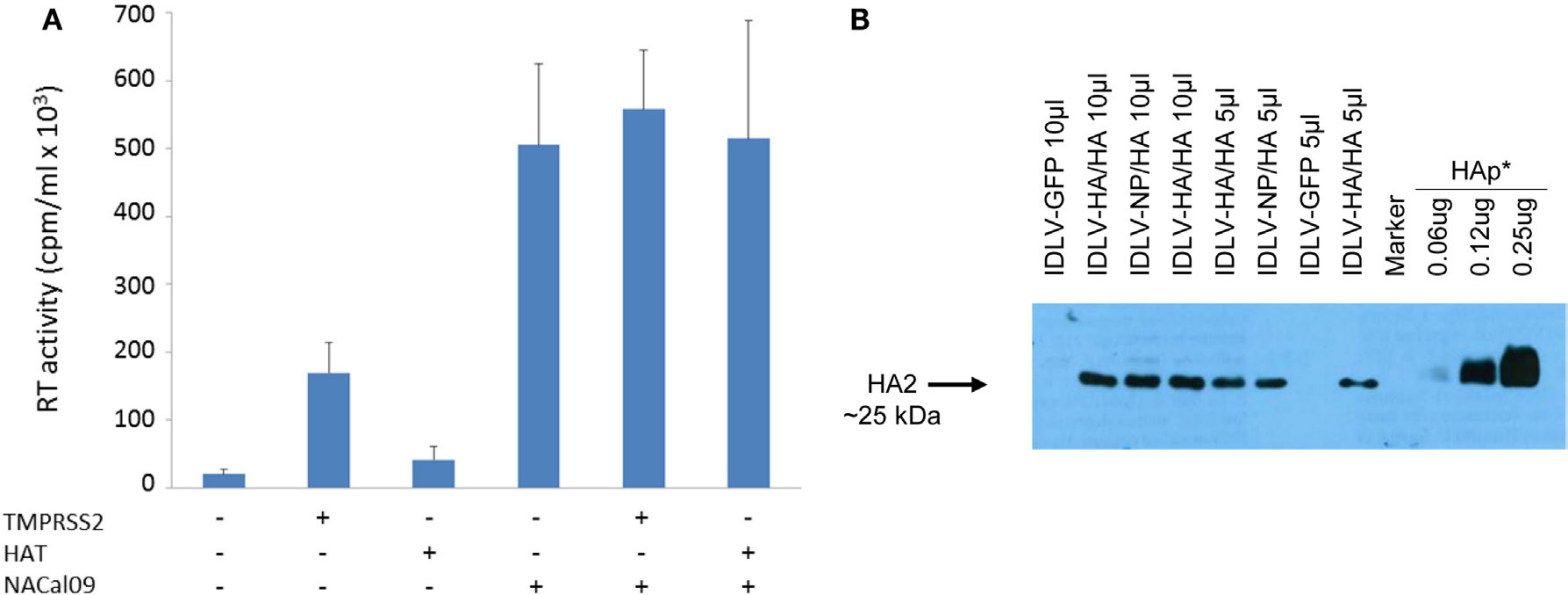
Production and validation of integrase defective lentiviral vector (IDLV) pseudotyped with hemagglutinin (HA). **(A)** Reverse transcriptase (RT) activity of IDLV expressing HA. Vectors were produced as described in Section “Materials and Methods” and in the presence of plasmids expressing transmembrane protease serine 2 (TMPRSS2), human airway trypsin (HAT), or NACal09, as indicated in the graph. Data are expressed as the mean result from three independent experiments. The error bars represent the standard errors of the mean. **(B)** Western blot (WB) of lysates from concentrated stocks of IDLV pseudotyped with HA showing incorporation of HA into IDLV. Note that IDLVs were produced in the presence of TMPRSS2 protease, resulting in the cleavage of HA0 to produce HA1 (not visualized here) and HA2. HA protein (HAp*, purified hemagglutinin vaccine subunit from influenza virus H1N1 A/California/7/2009) and IDLV-GFP were used as positive and negative control, respectively.

To produce IDLV expressing HA, 293 T LentiX cells were transfected with IN defective packaging plasmid, expressing the proteins required for IDLV assembly and release, the pseudotyping VSV.G-expressing plasmid, essential for IDLV entry into target cells, and HA-expressing lentiviral transfer vector plasmid, with the aim of pseudotyping the recombinant IDLV particles with the membrane tethered HA. Importantly, the presence of HA protein on the surface of the transfected cells was confirmed by flow cytometry and confocal microscopy (Figures 1A,B). However, the recovery of IDLV in the supernatant of the transfected cells was very low, indicating inhibition of release of IDLV pseudotyped with HA envelope (Figure 2A, left column). This was expected since, as with wild type influenza virus, NA protein is required for release of lentiviral vectors pseudotyped with influenza HA (17, 18, 48). To improve production of HA-pseudotyped IDLV, we cotransfected plasmids expressing NA protein, required for cleavage of surface sialic acid molecules on producer cells allowing release of the vector in the supernatant, and human serine transmembrane protease TMPRSS2 or HAT proteins, mediating proteolytic activation of influenza HA (18).

As shown in Figure 2A, recovery of IDLV-HA increased an average of eightfold in presence of TMPRSS2 but only twofold in the presence of HAT. Importantly, NA was fundamental for IDLV-HA release, increasing the amount of IDLV recovered in the supernatant an average of 24-fold over the IDLV produced in the absence of NA expressing plasmid. The inclusion of TMPRSS2 or HAT in addition to NA protein further improved, although not significantly, vector production (27-fold and 25-fold, respectively, compared to IDLV produced in the absence of NA and proteases). For producing concentrated IDLV preparations for immunization, TMPRSS2 was chosen over HAT protease expressing plasmid since IDLV production was generally higher and more consistent.

To produce IDLV expressing NP, vector was produced as described above and in the Section “Materials and Methods,” by including a NP-expressing lentiviral transfer vector plasmid and a plasmid expressing the HA protein for pseudotyping the IDLV produced in the 293 T cells. In Figure 2B, is shown a representative western blot (WB) analysis of lysates from concentrated stocks of IDLV showing the presence of HA protein in all purified IDLV preparations.

### A Single Immunization with IDLV Induces High and Persistent Levels of Binding and Antiviral Neutralizing Antibodies

**Figure 3:**
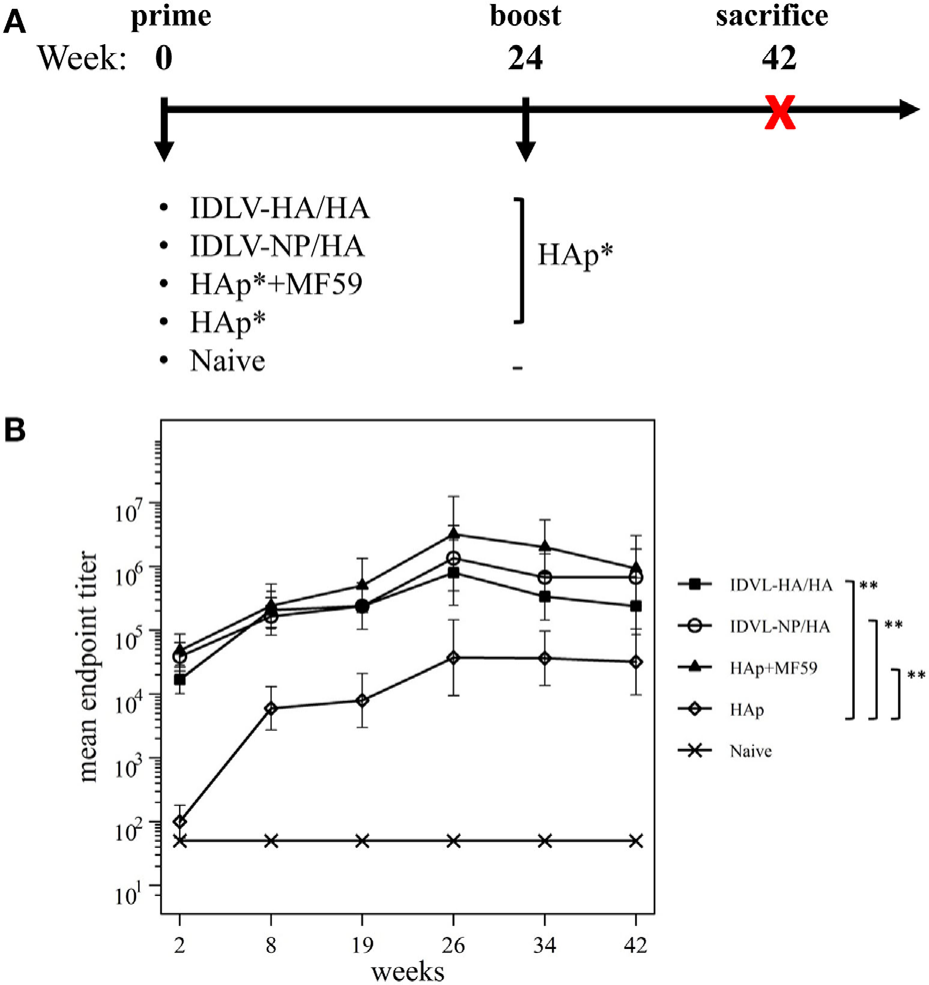
Immunization schedule and kinetics of anti-H1N1 binding antibodies (Abs). **(A)** Immunization schedule. CB6F1 mice (four mice/group) were primed once intramuscularly with integrase defective lentiviral vector (IDLV) expressing hemagglutinin (HA) and pseudotyped with HA (IDLV-HA/HA), IDLV expressing NP and pseudotyped with HA (IDLV-NP/HA), HA protein (*purified hemagglutinin vaccine subunit from influenza strain H1N1 A/California/7/2009) in combination with MF59 as an adjuvant (HAp + MF59), HA protein alone (HAp) or left untreated (Naive). All groups except for naive were boosted with HAp at 24 weeks after the prime. Blood was collected at several time points in order to perform ELISA and IFNγ ELISPOT assays. **(B)** Kinetics of serum anti-H1N1 IgG Abs. Serum samples from all groups were collected at the indicated time points after the prime and were assayed for the presence of anti-H1N1 IgG by ELISA. Results are expressed as predicted mean endpoint titers. The predicted mean values at each time point were estimated by a system of piecewise linear regressions including all antibody measurements within a structural equation modeling (SEM) frame work. Error bars indicate the 95% confidence interval. Asterisks indicate significant differences between groups at all the analyzed time points; **p-value <0.01.

To mimic the immunization protocol of the seasonal influenza in humans, mice were given a single immunization and the immune responses were analyzed at various time points up to 24 weeks. As positive controls, groups of mice were immunized with H1N1 subunit vaccine alone (HAp) or in combination with MF59 (HAp + MF59). The schedule of immunization is described in Figure 3A.

Humoral response to H1N1 was assessed initially by ELISA. As shown in Figure 3B, 2 weeks after the prime all vaccinated animals developed anti-H1N1 IgG antibodies in serum. The titers increased and persisted in all groups starting from 8 weeks after immunization. The highest titers were recovered in animals vaccinated with MF59, while the lowest levels were detected in the HAp group. No significant difference was observed between HAp + MF59 group and IDLV groups. Importantly, in both groups of IDLV vaccinated animals sustained Abs were induced at significantly higher titers than HAp alone (*p* < 0.01).

To assess the recall response, mice were boosted with HAp alone 6 months after the prime. Two weeks after the immunization with HAp all groups showed a boost in terms of binding Ab titers (Figure 3B). In particular, IDLV vaccinated animals were able to increase the anti-H1N1 Abs when boosted with HAp, indicating that immunization with IDLV enables the animals to respond to the HA antigen delivered in a different way, as a subunit vaccine in this case. The responses were persistent up to 18 weeks after the boost in all vaccinated animals. After the boost the differences between IDLV immunized animals and HAp group remained statistically significant (*p* < 0.01). Again, no significant difference in Ab titers between both IDLV groups and the adjuvanted group was seen.

**Figure 4:**
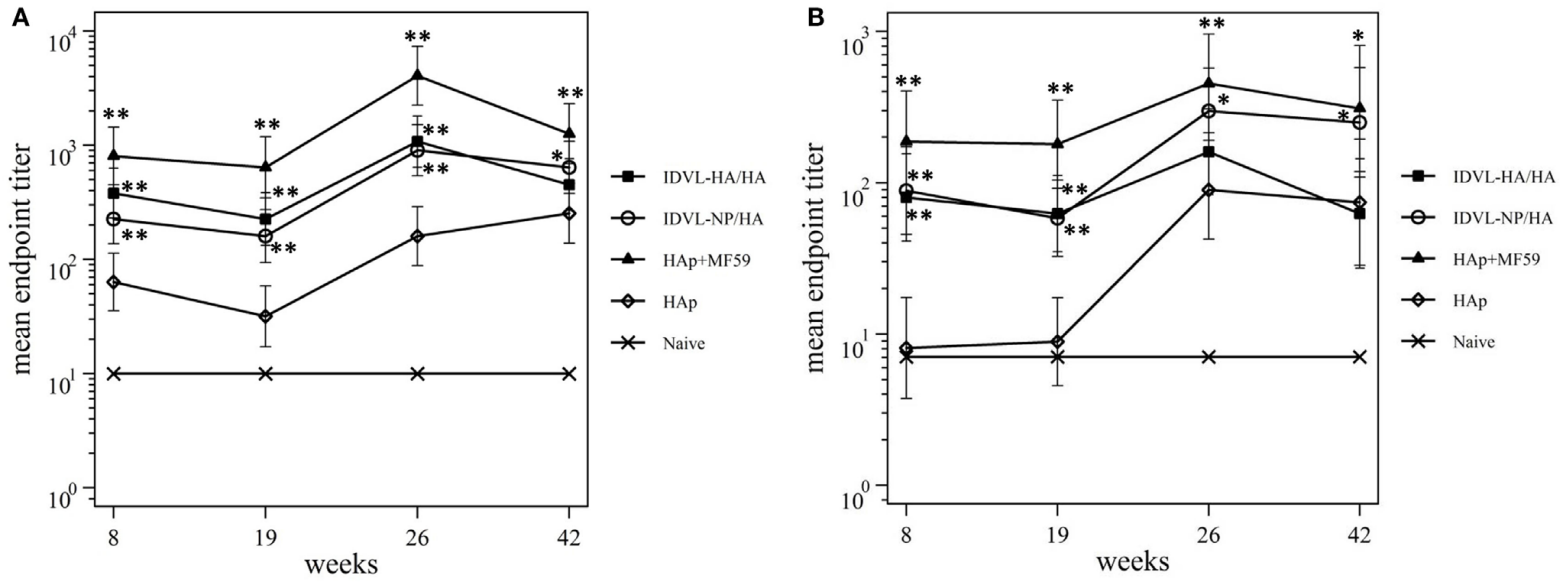
Kinetics of functional antihemagglutinin antibodies (Abs). Serum samples from all groups were collected at the indicated time points after the prime and were assayed for the presence of neutralizing Abs against A/California/7/09 virus, by hemagglutination inhibition assay **(A)** and microneutralization assay **(B)**. Results are expressed as predicted mean endpoint titers. The predicted mean values at each time point were estimated by a system of piecewise linear regressions including all antibody measurements within a structural equation modeling frame work. Error bars indicate the 95% confidence interval. Asterisks indicate significant differences compared to the HAp group, at the indicated time points; **p < 0.01; *p < 0.05.

To assess the functional antibodies, HAI assay, and microneutralization (MN) assay were performed at 8 and 19 weeks post priming and 2 weeks and 18 weeks post boost (Figure 4). HAI titers were present in all groups at 8 weeks postimmunization and were still detectable at 19 weeks post priming (Figure 4A). The response significantly increased after the boost in all groups. The kinetics of HAI titers mirrored the kinetics of binding Abs, showing the highest titers in the adjuvant treated group and the weakest ones in the HAp immunized animals. Both groups of IDLV vaccinated animals showed similar levels of HAI titers (*p* > 0.05), significantly lower than HAp + MF59 (*p* < 0.05), but significantly higher compared to HAp vaccinated animals (*p* < 0.01). Interestingly, the MN assay showed absence of neutralizing activity in the HAp alone group of mice after the prime, while MN titers were always present in both groups of IDLV vaccinated animals (Figure 4B). Again the highest activity was present in serum samples from HAp + MF59 immunized group. The boost increased the MN titers in all groups, including the HAp immunized animals.

### IDLV Induces Anti-NA Response

**Figure 5:**
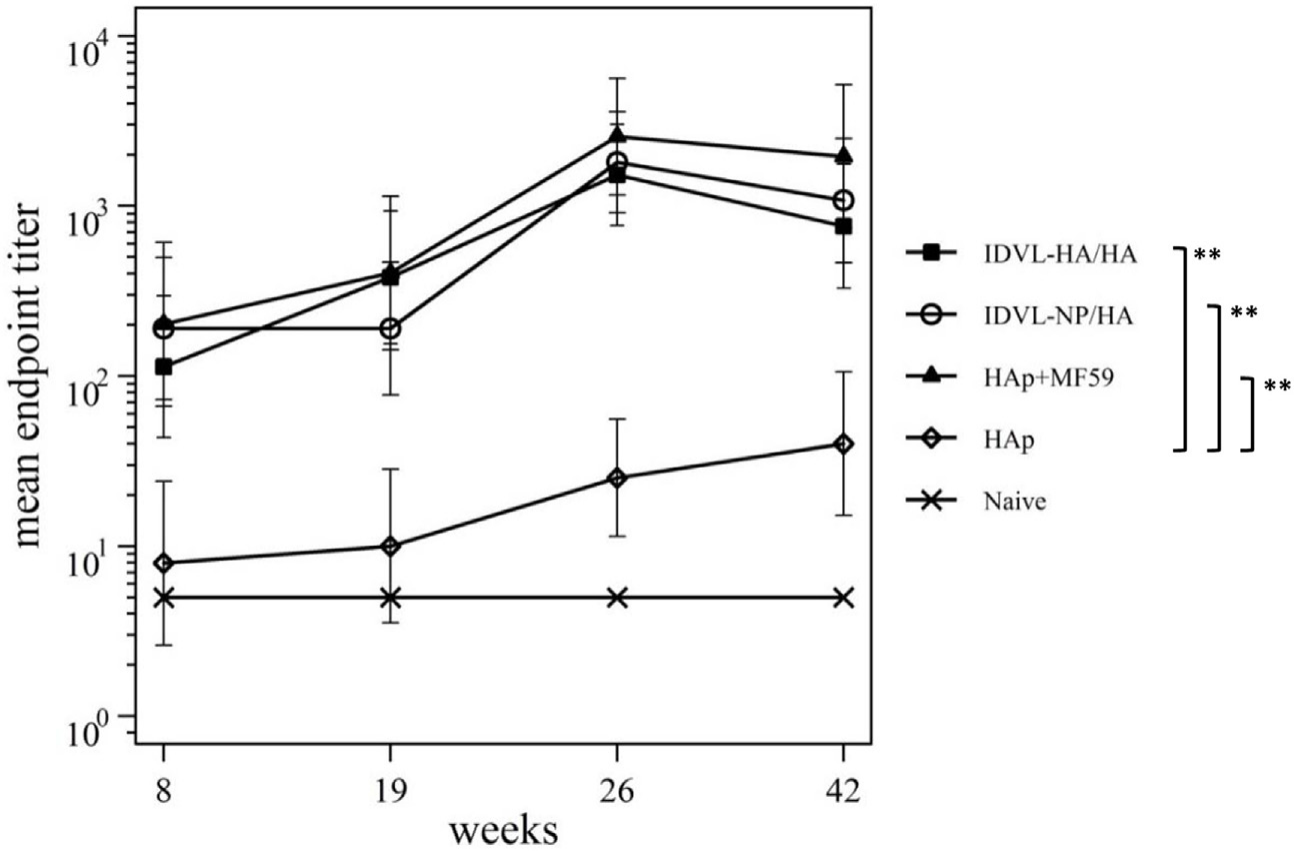
Kinetics of functional anti-neuraminidase (anti-NA) antibodies (Abs). Serum samples from all groups were collected at the indicated time points after the prime and were assayed for the presence of anti-NA Abs by ELLA assay (see Materials and Methods). Results are expressed as predicted mean endpoint titers. The predicted mean values at each time point were estimated by a system of piecewise linear regressions including all antibody measurements within a structural equation modeling frame work. Error bars indicate the 95% confidence interval. Asterisks indicate significant differences compared to the HAp group at all the analyzed time points; **p–value <0.01.

Since NA plasmid was included during the IDLV preparation and NA protein is present in subunit vaccine preparations (49, 50), we investigated the anti-NA response in all groups of immunized animals. Serum samples were thus assayed for NI activity. As shown in Figure 5, both IDLV groups showed a strong NI activity at all indicated time points after the prime that was significantly boosted after the immunization with HAp. A similar response was detected in the adjuvant vaccinated group, while the animals vaccinated with HAp alone did not generate detectable anti-NA response after the prime, showing low NI activity only after the boost.

### IDLV Vaccination Induces T Cell Response to HA and NP Delivered as Transgenes

**Figure 6:**
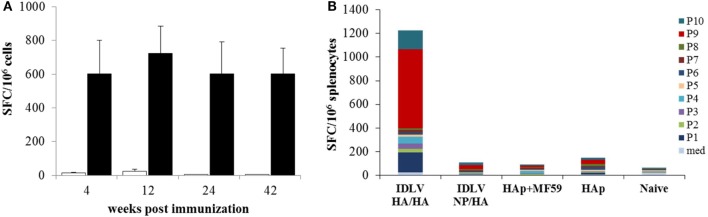
Analysis of antigen-specific T cells response. **(A)** Kinetics of nucleoprotein (NP)-specific T cell response in mice immunized with IDLV-NP/HA. IFNγ ELISPOT was performed using blood cells collected at 4, 12, and 24 weeks post-prime and splenocytes collected at 42 weeks post-prime. Results are expressed as mean spot forming cells (SFC) per 10^6^ cells. Cells were stimulated overnight with the MHC I-restricted epitope derived from NP protein sequence (black bars) or left untreated (white bars). Error bars indicate the SD among mice of the same group. **(B)** Analysis of HA-specific T cell response in immunized mice. IFNγ ELISPOT was performed using splenocytes collected at 42 weeks post-prime, using 10 pools of 15mers spanning the full length of HA protein, as described in Section “Materials and Methods.” Results are expressed as cumulative mean SFC per 10^6^ cells for each pool among mice of the same indicated group.

In order to assess the NP specific CD8-restricted cellular response in mice immunized with IDLV-NP/HA, INFγ ELISPOT was performed using blood cells collected at 4, 12, and 24 weeks after the prime, stimulated with MHC Class I-restricted NP peptide (Figure 6A). As expected, a high number of IFNγ producing T cells was detected in animals vaccinated with IDLV-NP/HA overtime, confirming a strong and persistent CD8+ T cell response directed to the transgene delivered by IDLV. The NP-specific T cell response was also analyzed in splenocytes at 42 weeks after a single IDLV-NP/HA immunization further confirming the persistence of transgene-specific IFNγ producing CD8^+^ T cells (Figure 6A).

In order to evaluate the HA specific response, INFγ ELISPOT was also performed in splenocytes from all groups of mice at sacrifice 18 weeks after the boost (42 weeks after the prime). Ten pools of 15mer peptides spanning the entire HA protein were used and the cumulative mean response against the HA pools is shown in Figure 6B. HA-specific IFNγ producing cells were detected only in mice primed with IDLV-HA/HA, while no positive response was observed in mice immunized with HAp with or without the adjuvant. In particular, pool 1, pool 9, and pool 10 (mean of 165.4, 666.2, 158.1 SFC/10^6^ cells, respectively) were responsible for the HA-specific response.

## Discussion

In this study, we assessed the feasibility of improving the strength of immune response for influenza vaccination by administering IDLV engineered to express either HA or NP proteins as transgenes, for induction of T cell responses and to carry HA on the surface of IDLV particles for a concomitant induction of functional antibodies against influenza virus.

Immunization with either HA-pseudotyped IDLV induced functional and durable Ab responses that were further increased after boosting with H1N1 purified subunit vaccine. This suggests that for induction of HA-specific Abs, HA protein does not need to be expressed from the IDLV transgene. Titers were lower or comparable to those obtained after immunization with MF59-adjuvanted H1N1 purified subunit vaccine, which we used as a gold standard for the induction of a strong and effective antibody response against influenza virus in mice. In particular, persistent HAI titers and anti-HA neutralizing Abs against homologous influenza virus strain were detected throughout the time course after the prime and were further increased after the boost. Of note, mice immunized with IDLV-HA/HA and IDLV-NP/HA induced functional anti-NA Abs, which were detected after the prime and increased after the boost, as measured by the NI assay. Titers were comparable to those obtained after immunization with MF59-adjuvanted H1N1 purified subunit vaccine, but significantly higher compared to HAp immunized animals. NA activity is required for the release of HA-pseudotyped IDLV from producer cells and a plasmid expressing the NA protein was included during preparation of IDLV for enabling vector production, as described in other settings (18). Although NA-specific Abs may not effectively prevent viral infection in humans, they may inhibit virus spread and reduce the severity of disease (51, 52), and a recent report provided strong evidence that NI titers correlated more significantly with reduced disease severity in a healthy volunteer challenge study performed with a wild-type influenza A challenge virus (53). Additional characterization of IDLV vaccine-induced functional antibodies, such as mapping of broadly neutralizing antibodies directed to the stem region of HA, will provide further information on the value of our platform.

While induction of HA-specific antibodies represents the primary strategy for prevention and control of influenza after vaccination in humans (36), the highly conserved viral NP has become an important focus for the development of broad, cross-protective or “universal” influenza vaccines. NP-specific cell-mediated responses have been shown to be protective against homologous and heterosubtypic influenza virus challenge in animal models (13, 54–57) and may help in reducing disease severity through enhancing viral clearance in humans (30, 58). In our previous work (13), we assessed the ability of IDLV-NP to induce protective immunity using different routes of immunization. In particular, we demonstrated that intranasal administration of IDLV-NP was more efficient than intramuscular immunization in protecting mice from influenza virus challenge. Although the protection from influenza challenge in mice can be achieved in presence of NP-specific T cell responses, this correlation has not been confirmed in humans, where the T cell responses directed toward conserved proteins may instead help in controlling the disease symptoms (28–30). To further improve the strength of our vaccine and to render the platform more suitable for human use, in the present work we focused on the development of a new vector strategy for expressing influenza antigens not only as transgenes but also as surface molecules. Here we demonstrated that the multi antigen IDLV-based influenza vaccine induced durable and functional immune responses in terms of NP or HA transgene-specific T cell responses and protective antibodies directed toward the surface proteins HA and NA. In particular, NP-specific CD8-restricted cellular responses, as measured by INFγ ELISPOT, were present and maintained up to 42 weeks after a single immunization in mice immunized with IDLV-NP/HA. Similarly, HA-specific IFNγ producing cells were detected in mice immunized with IDLV-HA/HA, but not in mice immunized with HAp with or without MF59 adjuvant.

Other approaches have been tested for delivering multiple antigens for inducing cellular and humoral immunity. As an example, a recent report using Adenovirus and MVA delivering Influenza NP, M1 and HA antigens in a prime-boost regimen showed induction of immune responses against the delivered antigens and protection after challenge (59). The approach described in our report shows that multivalent IDLVs efficiently induce a prolonged cellular immune response due to expression of the vectored HA and NP transgenes from episomal DNA circles, as described in several settings (1, 2) and functional humoral responses to HA and NA proteins.

Previous work has shown that recombinant influenza vaccines containing insect cell-expressed virus-like particles (VLPs) displaying H1N1 may constitute a promising vaccination approach (60). Pseudotype-based influenza genes delivery represents an alternative to successfully express HA in mammalian cells providing an efficacious vaccine when tested in chickens and mice (61, 62). More recently, Venereo-Sanchez et al. showed that HIV-Gag-based VLP displaying H1N1 induced a sustained immune response which provided full protection after lethal challenge with the homologous virus strain in mice (63). Of note, IDLVs used in our report were pseudotyped with VSV.G glycoprotein which may also contribute to the strength of the IDLV-induced immune responses by increasing the tropism of the vector. In fact, it has been shown that HIV VLP pseudotyped with VSV-G are more immunogenic compared to VLP that lacked VSV-G, in a monkey model of immunization (64) and that VSV-G-pseudotyped LV can adhere to transduced cells for a substantial amount of time, thus leading to additional cycles of transduction (65–67). These are potentially important advantages for exploiting recombinant IDLV pseudotyped with VSV.G and/or other heterologous viral proteins.

In conclusion, our study highlights the potential for IDLV to be developed for use as a novel multiantigen vaccine platform against influenza virus. Combination of NP, HA, and NA antigens in the same IDLV results in a more comprehensive and functional immune response, which may be beneficial to prevent and/or control influenza virus infection.

## Ethics Statement

Animals were maintained under specific pathogen-free conditions in the animal facilities at the Istituto Superiore di Sanità (ISS) and treated according to European Union guidelines and Italian legislation (Decreto Legislativo 26/2014). All animal studies were authorized by the Italian Ministry of Healthy and reviewed by the Service for Animal Welfare at ISS (Authorization n. 314/2015-PR of 30/04/2015). All animals were euthanized by CO2 inhalation using approved chambers, and efforts were made to minimize suffering and discomfort.

## Author Contributions

AG, AC, and DN designed the experiments, analyzed the data, and wrote the article; AG, MB, RB, FG, LC, LP, SC, DM, VM, AC, and ZM performed experiments; MV performed statistical analysis; MC analyzed the data and critically edited the manuscript; NT provided technical knowhow on pseudotype production and critically edited the manuscript; MS provided key reagents and critically edited the manuscript. All authors have contributed to the drafting of the manuscript, have revised the work, and have approved the final version.

## Conflict of Interest Statement

The authors declare that the research was conducted in the absence of any commercial or financial relationships that could be interpreted as a potential conflict of interest.
